# Coinfection of Porcine Circovirus 2 and Pseudorabies Virus Enhances Immunosuppression and Inflammation through NF-κB, JAK/STAT, MAPK, and NLRP3 Pathways

**DOI:** 10.3390/ijms23084469

**Published:** 2022-04-18

**Authors:** Xue Li, Si Chen, Liying Zhang, Guyu Niu, Xinwei Zhang, Lin Yang, Weilong Ji, Linzhu Ren

**Affiliations:** College of Animal Sciences, Key Lab for Zoonoses Research, Ministry of Education, Jilin University, 5333 Xi’an Road, Changchun 130062, China; lixue9915@mails.jlu.edu.cn (X.L.); sichen20@mails.jlu.edu.cn (S.C.); zhangliy@jlu.edu.cn (L.Z.); niugy9916@mails.jlu.edu.cn (G.N.); xwzhang17@mails.jlu.edu.cn (X.Z.); linyang20@mails.jlu.edu.cn (L.Y.); jiwl19@mails.jlu.edu.cn (W.J.)

**Keywords:** porcine circovirus 2 (PCV2), pseudorabies virus (PRV), infection, immune response

## Abstract

Porcine circovirus 2 (PCV2) and pseudorabies virus (PRV) are economically important pathogens in swine. PCV2 and PRV coinfection can cause more severe neurological and respiratory symptoms and higher mortality of piglets. However, the exact mechanism involved in the coinfection of PRV and PCV2 and its pathogenesis remain unknown. Here, porcine kidney cells (PK-15) were infected with PCV2 and/or PRV, and then the activation of immune and inflammatory pathways was evaluated to clarify the influence of the coinfection on immune and inflammatory responses. We found that the coinfection of PCV2 and PRV can promote the activation of nuclear factor-κB (NF-κB), c-Jun N-terminal protein kinases (JNK), p38, and nod-like receptor protein 3 (NLRP3) pathways, thus enhancing the expression of interferon-γ (IFN-γ), interferon-λ1 (IFN-λ1), interferon-stimulated gene (ISG15), interleukin 6 (IL6), and interleukin 1β (IL1β). Meanwhile, PCV2 and PRV also inhibit the expression and signal transduction of IFN-β, tumor necrosis factor α (TNFα), and the Janus kinase-signal transducer and activator of transcription (JAK/STAT) pathway. In addition, PCV2 and PRV infection can also weaken extracellular-signal-regulated kinase (ERK) activity. These results indicate that the regulations of cellular antiviral immune responses and inflammatory responses mediated by NF-κB, JAK/STAT, mitogen-activated protein kinase (MAPK), and NLRP3 pathways, contribute to immune escape of PCV2 and PRV and host antiviral responses.

## 1. Introduction

Porcine circovirus 2 (PCV2) is the causative agent of porcine circovirus diseases and porcine circovirus-associated diseases (PCVD/PCVAD), which are distributed worldwide, and the positive rate of pigs infected with PCV2 is high, or even more than 90% [1,2]. However, more and more evidence shows that a single infection with PCV2 will not lead to obvious cytopathic effect (CPE) in vitro and clinical disease in vivo [2,3,4], and most single infection cases of PCV2 result in subclinical symptoms with significant immunosuppression in pigs [2,5]. Subsequently, the immunosuppression induced by PCV2 infection is beneficial to the secondary infection of other pathogens. Therefore, coinfections of PCV2 with other pathogens, including viruses and bacteria, etc., become the main factors causing severe diseases in pigs [2,4].

Among pathogens co-infected with PCV2, viruses are the most common causes of severe diseases. As reported, the coinfection rates of PCV2 with porcine reproductive and respiratory syndrome virus (PRRSV), pseudorabies virus (PRV), classical swine fever virus (CSFV), and porcine epidemic diarrhea virus (PEDV) were 26.73%, 18.37%, 13.06%, and 3.47%, respectively, in Shandong province in China from 2015 to 2018 [6]. It is worth noting that coinfection of PCV2 with type 1 modified live vaccine-like PRRSV strain (MLV1-like) resulted in increased virulence in the infected pigs [7]. Sequential infection of piglets with highly pathogenic PRRSV and PCV2 exhibited synergistic effects and more severe clinical symptoms and lesions on piglets [8]. However, so far, only a few studies have focused on the pathogenesis of PCV2 and PRV coinfection.

PRV (also named Suid herpesvirus 1) belongs to the genus *Varicellovirus*, *Alphaherpesvirinae* subfamily, the family *Herpesviridae*, which mainly causes neurological symptoms and high mortality of piglets, growth retardation, and respiratory disorders in growing pigs, and reproductive failure of sows [9]. Although PRV infection has been successfully controlled in several countries, PRV is still one of the most important pathogens of swine and wild boar all over the world, especially the highly pathogenic PRV that has emerged in recent years, due to the recombination between wild PRV and the attenuated live vaccine strain [1,3,10,11].

It was reported that PCV2 can promote the expression of interleukin 6 (IL6), IL8, IL10, pro-inflammatory cytokines, and activate nuclear factor-κB (NF-κB), thus coordinating cellular immune response and inflammatory reaction, and promoting virus infection [12,13,14]. PCV2 inhibits interferon-β (IFN-β) through the p38-mitogen-activated protein kinase (MAPK) pathway [15] but also enhances IFN-β via retinoic acid-inducible gene I (RIG-1) and interferon regulatory factor 7 (IRF7) signaling pathways [16]. Moreover, PCV2 infection can induce immunosuppression in pigs, but external immuno-stimulation, such as vaccination and coinfection, has been proved to aggravate PCVAD [13]. Furthermore, PRV infection triggers persistent and aberrant NF-κB activation through DNA damage response but inhibits NF-κB-dependent gene expression [17,18]. PRV also activates cytokine storms by upregulating the expression levels of IFN-α, IFN-β, tumor necrosis factor α (TNFα), IL1β, IL6, and IL18, and stimulates the pyroptosis pathway, by enhancing the expression levels of nod-like receptor protein 3 (also named NACHT, LRR, and PYD domains-containing protein 3, NLRP3), caspase-1, Gasdermin-D, and IL1β/18 [19]. Virulent PRV induces specific and fatal systemic inflammation through two main cytokines IL6 and granulocyte colony-stimulating factor (G-CSF) [20]. Notably, coinfection of PCV2 and PRV caused more severe neurological and respiratory symptoms and higher mortality of piglets, with an increased PRV replication in brain and lung tissues [3]. PCV2 and PRV coinfection is also considered the main cause of porcine respiratory disease complex (PRDC) [2]. PCV2 infection can lead to interleukin 10 (IL-10)- mediated immunosuppression [21,22], which may impair immune responses against PRV, resulting in more severe disease or vaccination failure against PRV [21,22,23,24]. However, the exact mechanism involved in the coinfection of PRV and PCV2, and its pathogenesis, remain unknown. Therefore, it is necessary to analyze the regulatory mechanism of PCV2 and PRV coinfection on immune response and inflammatory reaction, so as to provide a theoretical basis, and effective targets, for the prevention and control of related diseases. In the present study, porcine kidney cells (PK-15) were infected individually or co-infected with PCV2 and/or PRV, followed by an evaluation of the immune and inflammatory pathways involved.

## 2. Results

### 2.1. Coinfection of PCV2 and PRV Inhibit Each Other

**Figure 1:**
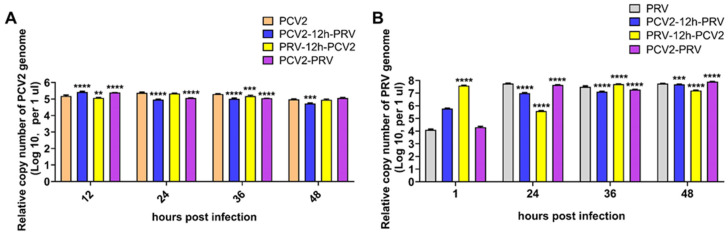
**Coinfection of PCV2 and PRV affects in PK-15 cells.** Viral genomic copies were evaluated using real-time PCR at indicated hour post-infection. The data are presented as the means ± SD. **, *p*-value < 0.01; ***, *p*-value < 0.001; ****, *p*-value < 0.0001. (**A**) PCV2. (**B**) PRV.

To evaluate the replication of PCV2 and PRV, PK-15 cells were infected with different combinations of PCV2 and PRV, including PCV2 or PRV single infection, PCV2 and PRV coinfections (PCV2+PRV group, PCV2-12h-PRV group, and PRV-12h-PCV2 group). As shown in Figure 1, all PCV2 and/or PRV infected groups can replicate in PK-15 cells, suggesting that coinfection of PCV2 and PRV is feasible in vitro. Furthermore, the growth curves of PCV2 and PRV in the coinfection groups were similar. Compared with the single infection, the copy number of PCV2 in the coinfection group decreased at 24 to 48 hpi, suggesting that PRV may inhibit the proliferation of PCV2 (Figure 1A). Compared with PRV infection alone, the coinfection group PCV2-12h-PRV can inhibit PRV proliferation (Figure 1B). The growth curves of PRV in the coinfection groups (PCV2+PRV group, PRV-12h-PCV2 group) were similar to that of the PRV single infection group.

### 2.2. Coinfection of PCV2 and PRV Inhibits the Expression of IFN-β, IRF3, and ISG56/IFIT1 but Promotes the Expression of IFN-γ, IFN-λ1, IRF7, and ISG15

**Figure 2:**
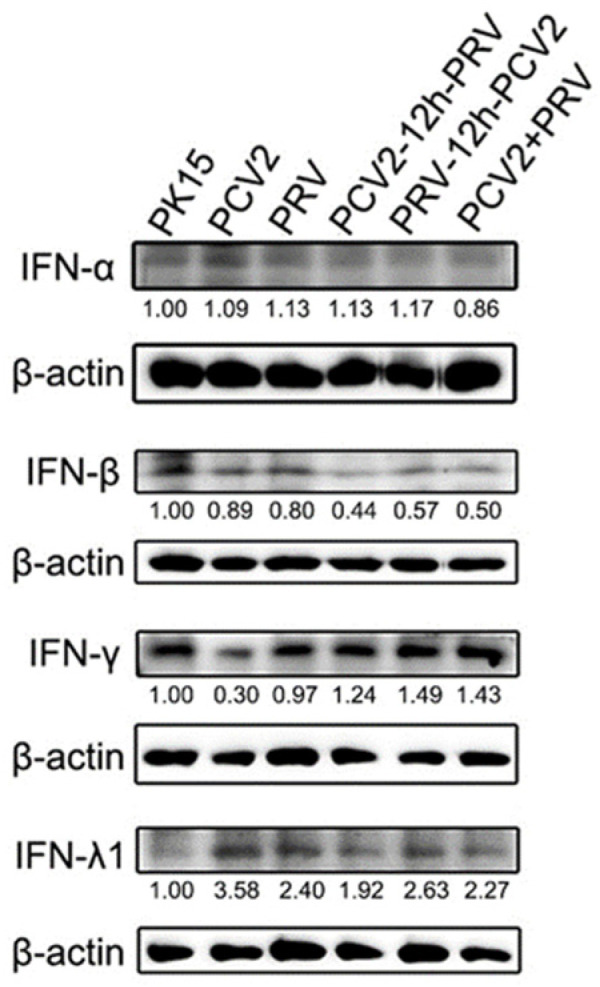
Coinfection of PCV2 and PRV modulates IFNs. Western blotting was performed using Interferon alpha 1 Antibody, Interferon beta/IFNB, IFN-γ, IL28/29 (H-1), and Anti-β-Actin Antibody as primary antibodies, respectively. HRP-labeled Goat Anti-mouse IgG (H+L) and HRP-labeled Goat Anti-rabbit IgG (H+L) were used as the secondary antibody. β-actin was used as a control. The average expression level of the target protein in each group is shown below each lane. The protein amount of the PK-15 group is set to 1, and the values of other groups are the ratio with the PK-15 group. Unprocessed original images can be found in Supplementary Figure S1.

IFNs are important components of the innate immune response against virus infection. Therefore, the expression levels of IFNs were detected by Western blot. As shown in Figure 2, the levels of IFN-α in the infected groups had no significant difference compared with the control group. On the contrary, levels of IFN-β showed a decrease in the infected groups, especially in the co-infected groups (Figure 2). Levels of IFN-γ were decreased in cells infected with PCV2 or PRV alone but increased in the coinfection groups (Figure 2). Notably, levels of IFN-λ1 were enhanced in all the infected groups compared with the control group (Figure 2).

**Figure 3:**
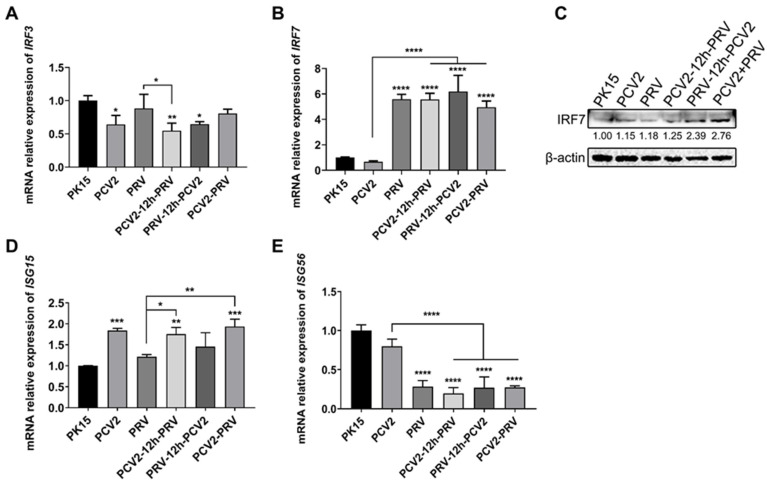
**Coinfection of PCV2 and PRV regulates IRFs.** (**A**,**B**) Expression levels of IRFs. The mRNA levels of *IRF3* (**A**) and *IRF7* (**B**) were evaluated via real-time PCR. (**C**) Protein levels of IRF7. Western blotting was performed using Rabbit Anti-IRF7 antibody and Anti-β-Actin Antibody as primary antibodies, respectively. HRP-labeled Goat Anti-mouse IgG (H+L) and HRP-labeled Goat Anti-rabbit IgG (H+L) were used as the secondary antibody. β-actin was used as a control. The average expression level of the target protein in each group is shown below each lane. The protein amount of the PK-15 group is set to 1, and the values of other groups are the ratio with the PK-15 group. (**D**,**E**) Expression levels of ISGs. The mRNA levels of *ISG15* (**D**) and *ISG56* (**E**) were evaluated via real-time PCR. *, *p*-value < 0.05; **, *p*-value < 0.01; ***, *p*-value < 0.001; ****, *p*-value < 0.0001. The data are presented as the means ± SD. Unprocessed original images can be found in Supplementary Figure S2.

IRFs, especially IRF3 and IRF7, are members of the interferon regulatory factor family, which are closely related to the expression of interferon genes during virus infection [25,26]. Compared with the control group, the expression of the *IRF3* gene in all groups with PCV2 was inhibited, whereas PRV infection alone does not affect *IRF3* expression (Figure 3A), indicating PCV2 may be the dominant factor for IRF3 inhibition, thus inhibiting IRF3-related immune response.

Compared with the control group, expression levels of the *IRF7* gene were enhanced in all groups containing PRV, including single- and co-infected groups, but there was no difference in the PCV2 single-infected group (Figure 3B). These results suggest that coinfection of PCV2 and PRV induces immune responses by activating IRF7. To further confirm these results, Western blotting was performed to examine the protein levels of IRF7. As shown in Figure 3C, levels of IRF7 were enhanced in the virus-infected groups compared to that of the control group, which is more obvious in PRV-containing groups. Therefore, the down-regulation of IRF3 expression is mainly caused by PCV2 infection, whereas PRV mainly modulates the expression of IRF7. The coinfection of the two viruses can obviously up-regulate IRF7 in cells. 

Moreover, expression levels of *IFN-stimulated gene 15 (ISG15)* were increased in cells infected with PCV2 alone compared with that of the control group (Figure 3D), whereas no obvious difference in *ISG56/IFIT1* gene was observed in the cells infected with PCV2 alone (Figure 3E). The expression level of the *ISG56* gene was significantly decreased in cells infected with PRV alone than in the control group (Figure 3E), whereas no obvious difference in the *ISG15* gene was observed in the cells infected with PRV alone (Figure 3D). Expression levels of the *ISG15* gene in the co-infected groups were up-regulated compared with the PRV group, while expression levels of the *ISG56* gene in the co-infected groups were down-regulated compared with the PCV2 group (Figure 3D,E). 

These results indicate that coinfection of PCV2 and PRV inhibited the expression of IRF3, IFN-β, and ISG56, but promoted the expression of IRF7, IFN-γ, IFN-λ1, and ISG15.

### 2.3. Coinfection of PCV2 and PRV Suppress JAK1- and STAT1-Related JAK/STAT Pathways 

**Figure 4:**
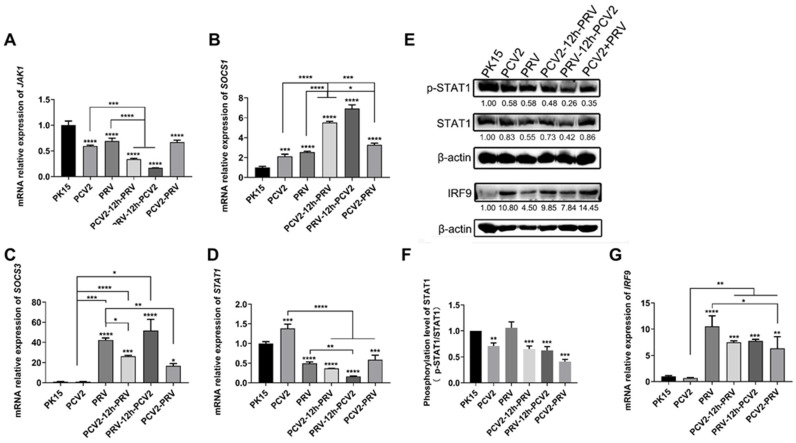
Coinfection of PCV2 and PRV suppresses STAT1-related JAK/STAT pathways. (**A**–**D**) Expression levels of JAK/STATs. The relative mRNA levels of *JAK1* (**A**), *SOCS1* (**B**), *SOCS3* (**C**), and *STAT1* (**D**) were detected using real-time PCR. (**E**,**F**) Protein levels of STAT1 and IRF9. Western blotting was performed using p-STAT1, Anti-STAT1 Antibody, IRF9 Antibody, and Anti-β-Actin Antibody as primary antibodies, respectively. HRP-labeled Goat Anti-mouse IgG (H+L) and HRP-labeled Goat Anti-rabbit IgG (H+L) were used as the secondary antibody. β-actin was used as a control. The average expression level of the target protein in each group is shown below each lane. The protein amount of the PK-15 group is set to 1, and the values of other groups are the ratio with the PK-15 group. (**G**) Expression levels of *IRF9* were detected using real-time PCR. The data are presented as the means ± SD. *, *p*-value < 0.05; **, *p*-value < 0.01; ***, *p*-value < 0.001; ****, *p*-value < 0.0001. Unprocessed original images can be found in Supplementary Figure S3.

The Janus kinase-signal transducer and activator of transcription (JAK/STAT) pathway is one of the important signal pathways downstream of cytokine receptors [27]. IFN activates the JAK/STAT pathway, which then amplifies the IFN signal and modulates the expression of antiviral factors [27]. As shown in Figure 4, expression levels of the *JAK1* gene were significantly inhibited in all the virus-infected groups, including single and co-infected groups compared to that of the control group (Figure 4A), whereas expression levels of the *suppressors of cytokine signaling 1* gene (*SOCS1*) (Figure 4B), a negative regulator of the JAK/STAT signaling pathway, was significantly increased in the virus-infected groups. PCV2 infection alone does not affect the expression of the *SOCS3* gene, while PRV infection alone and coinfection of PCV2 and PRV significantly promoted the expression of the *SOCS3* gene (Figure 4C). Furthermore, although the expression of the *STAT1* gene was up-regulated in the PCV2 alone group, it was down-regulated significantly in other infected groups (Figure 4D). Moreover, the phosphorylation levels of STAT1 (p-STAT1/STAT1) were inhibited in all the coinfection groups (Figure 4E,F). In addition, compared with the control group, expression levels of the *IRF9* gene were enhanced in all groups infected with PRV, including single- and co-infected groups (Figure 4G). Furthermore, the expression levels of the IRF9 gene in coinfection groups were significantly lower than those in the PRV single-infected group (Figure 4G). These results further confirmed the results in Figure 4E, that the expressions of IRF9 were enhanced in the virus-infected groups compared to that of the control group (Figure 4E). 

Thus, coinfection of PCV2 and PRV can prevent the cascade of IFN signals by inhibiting the JAK1 and STAT1 (Figure 4). Coinfection of PCV2 and PRV leads to stronger immunosuppression on the IFN-JAK/STAT pathway than virus infection alone (Figure 2, Figure 3 and Figure 4).

### 2.4. Coinfection of PCV2 and PRV Modulates NF-κB Signal Pathway

**Figure 5:**
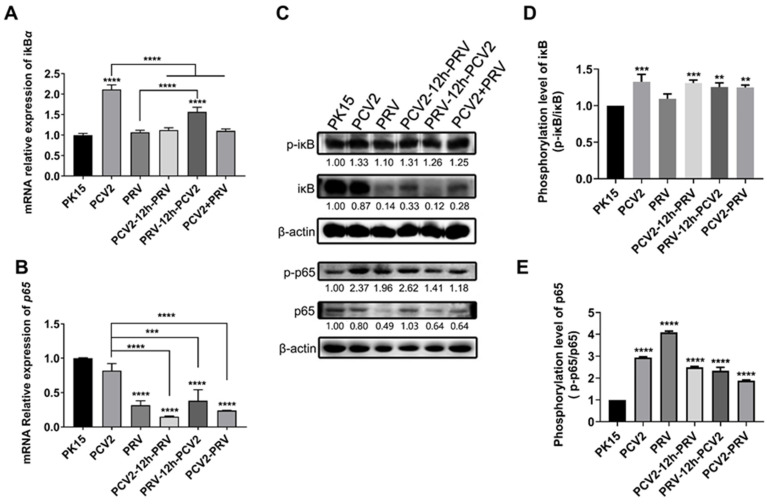
Coinfection of PCV2 and PRV modulates the NF-κB signal pathway. (**A**,**B**) Expression levels of *iκB* and *p65*. The relative mRNA levels of *iκB* (**A**) and *p65* (**B**) were examined using real-time PCR. The data are presented as the means ± SD. **, *p*-value < 0.01; ***, *p*-value < 0.001; ****, *p*-value < 0.0001. (**C**–**E**) Protein levels of p65 and iκB. Western blotting was conducted using P-IΚBα (Ser32 Ser36), IΚB-α, NFκB p65 Polyclonal Antibody, Phospho-NFκB p65 (Thr276) Polyclonal Antibody, and Anti-β-Actin Antibody as primary antibody, respectively. HRP-labeled Goat Anti-mouse IgG (H+L) and HRP-labeled Goat Anti-rabbit IgG (H+L) were used as the secondary antibody. β-actin was used as a control. The average expression level of the target protein in each group is shown below each lane. The protein amount of the PK-15 group is set to 1, and the values of other groups are the ratio with the PK-15 group. Unprocessed original images can be found in Supplementary Figure S4.

Nuclear factor-κB (NF-κB) plays a crucial role in regulating host immune responses and inflammation [28]. Therefore, levels of two NF-κB related factors, p65 and iκB, in PCV2 and/or PRV-infected cells were evaluated at 36 hpi via real-time PCR and western blotting. As shown in Figure 5A, compared with the control group, PRV infection alone and coinfection with PCV2 significantly inhibited the expression of the *p65* gene, while PCV2 infection alone had no significant effect on the expression of the *p65* gene. Furthermore, PCV2 infection alone and coinfection significantly promoted the expression of the *iκB* gene (Figure 5B). These results suggest that the single infection or coinfection of PRV and PCV2 may partially inhibit NF-κB at transcription levels. 

Compared with the control group, total proteins of p65 and iκB showed a slight or significant decrease in almost all the infected groups. However, levels of phosphorylated p65 (p-p65) and iκB (p-iκB) were significantly increased in the coinfected groups (Figure 5C–E). These results indicate that infection of PCV2 and PRV alone, or in combination, can stimulate the NF-κB signaling pathway at the translational level, and the cells infected with PRV exhibit more obvious stimulation of NF-κB signals.

### 2.5. Coinfection of PCV2 and PRV Modulates Expressions of Host Pro-Inflammatory Factors

**Figure 6:**
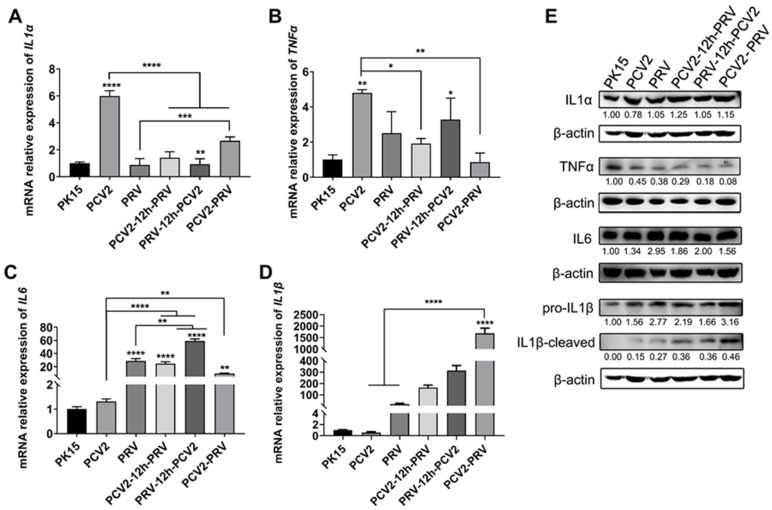
Coinfection of PCV2 and PRV modulates expressions of host pro-inflammatory factors. (**A**–**D**) Expression levels of pro-inflammatory factors. The relative mRNA levels of *IL1α* (**A**), *TNFα* (**B**), *IL6* (**C**), and *IL1β* (**D**) were evaluated using real-time PCR. The data are presented as the means ± SD. *, *p*-value < 0.05; **, *p*-value < 0.01; ***, *p*-value < 0.001; ****, *p*-value < 0.0001. (**E**) Protein levels of pro-inflammatory factors. Western blotting was performed using IL1α, IL1B Antibody, IL6, TNFα, and Anti-β-Actin Antibody as primary antibodies, respectively. HRP-labeled Goat Anti-mouse IgG (H+L) and HRP-labeled Goat Anti-rabbit IgG (H+L) were used as the secondary antibody. β-actin was used as a control. The average expression level of the target protein in each group is shown below each lane. The protein amount of the PK-15 group is set to 1, and the values of other groups are the ratio with the PK-15 group. Unprocessed original images can be found in Supplementary Figure S5.

Inflammation is a critical part of the host immune system’s response to infection. To assess the effect of viral infection on cellular inflammation, the expressions of several inflammatory factors were detected by real-time PCR. The results showed that PCV2 infection alone enhanced the expression levels of the *IL1α* gene (Figure 6A) and *TNFα* gene (Figure 6B), whereas PRV infection alone increased the expression of the *IL6* gene (Figure 6C). 

Compared with the PCV2 infection alone, coinfection of PCV2 and PRV decreased the expression levels of the *IL1α* gene (Figure 6A) and *TNFα* gene (Figure 6B) but increased the expression level of the *IL6* gene (Figure 6C) and *IL1β* gene (Figure 6D). Compared with the PRV infection alone, coinfection of PCV2 and PRV increased the expression levels of the *IL1α* gene (Figure 6A) and *IL1β* gene (Figure 6D), but decreased the expression level of the *IL6* gene (Figure 6C). The protein levels of IL6, and IL1β detected by Western blotting further confirmed these results (Figure 6E). However, the protein levels of TNFα in all infected groups were inhibited, and the inhibitions of TNFα in the coinfection groups were slightly stronger (Figure 6E), suggesting the virus may inhibit the expression of TNFα and then inhibit the aggravation of cellular inflammatory reaction. The above results further indicate that coinfection of PCV2 and PRV modulate expressions of host pro-inflammatory factors. The coinfection of PCV2 and PRV weakened the regulatory effect of a single virus infection on inflammation. 

**Figure 7:**
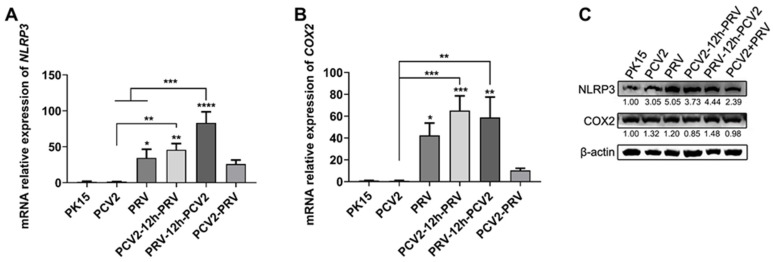
Coinfection of PCV2 and PRV activates the NLRP3 pathway. (**A**,**B**) Expression levels of NLRP3 and COX2. The relative mRNA levels of *NLRP3* (**A**) and *COX2* (**B**) were examined via Real-time PCR. The data are presented as the means ± SD. *, *p*-value < 0.05; **, *p*-value < 0.01; ***, *p*-value < 0.001; ****, *p*-value < 0.0001. (**C**) Protein levels of NLRP3 and COX2. Western blotting was performed using NLRP3, Cox-2, and Anti-β-Actin Antibody as primary antibodies, respectively. HRP-labeled Goat Anti-mouse IgG (H+L) and HRP-labeled Goat Anti-rabbit IgG (H+L) were used as the secondary antibody. β-actin was used as a control. The average expression level of the target protein in each group is shown below each lane. The protein amount of the PK-15 group is set to 1, and the values of other groups are the ratio with the PK-15 group. Unprocessed original images can be found in Supplementary Figure S6.

Inflammasomes regulate the activity of caspase-1 and the maturation of IL1β and IL-18, which act as innate immune system sensors and receptors in various infections, cancer, and other diseases, among which the NLRP3 inflammasome has been well characterized to date [29,30,31]. Therefore, the activity of the NLRP3 inflammasome in PRV and/or PCV2 infected cells was evaluated. As shown in Figure 7, the expression levels of the *NLRP3* gene (Figure 7A) and *cyclooxygenase 2* (*COX2/PTGS2*) gene (Figure 7B) were enhanced in all PRV- infected cells compared with that of the control group. Furthermore, the enhancement effects of NLRP3 and COX2 were significantly higher in the PRV single-infection group and two sequential infection groups than those in the coinfection group (PCV2+PRV). These results were further confirmed by Western blotting that the levels of NLRP3 and COX2 increased in all infected cells, especially NLRP3 (Figure 7C). Therefore, infection of PCV2 and PRV caused NLRP3-mediated cellular inflammatory reaction, and the coinfection of PCV2 and PRV aggravated the inflammation.

### 2.6. Coinfection of PCV2 and PRV Activates Inflammatory and Immune via p38 and JNK1/2

**Figure 8:**
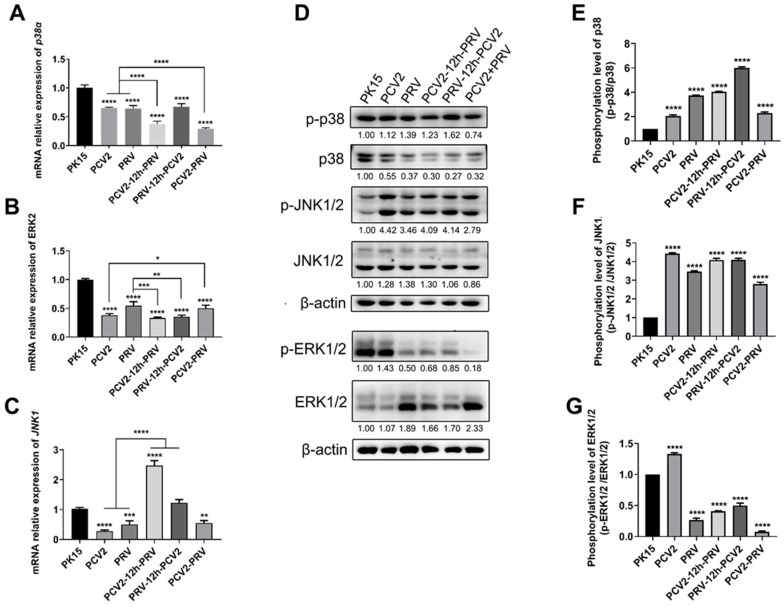
Coinfection of PCV2 and PRV activates inflammatory and immune responses via p38 and JNK1/2. (**A**–**C**) Expression levels of MAPKs. The relative mRNA levels of *p38* (**A**), *ERK1/2* (**B**), and *JNK1/2* (**C**) were examined via real-time PCR. The data are presented as the means ± SD. *, *p*-value < 0.05; **, *p*-value < 0.01; ***, *p*-value < 0.001; ****, *p*-value < 0.0001. (**D**–**G**) Protein levels of MAPKs. Western blotting was conducted using p-p38 (Thr180/Tyr182), P38, JNK (FL), p-JNK (Thr 183/Tyr 185), ERK p44/42 MAPK (Erk1/2) (137F5) Rabbit mAb, Phospho-p44/42 MAPK (Erk1/2) (Thr202/Tyr204) (D13.14.4E) XP^®^ Rabbit mAb, and Anti-β-Actin Antibody as primary antibody, respectively. HRP-labeled Goat Anti-mouse IgG (H+L), HRP-labeled Donkey Anti-Goat IgG (H+L), and HRP-labeled Goat Anti-rabbit IgG (H+L) were used as the secondary antibody. β-actin was used as a control. The average expression level of the target protein in each group is shown below each lane. The protein amount of the PK-15 group is set to 1, and the values of other groups are the ratio with the PK-15 group. Unprocessed original images can be found in Supplementary Figure S7.

The MAPK pathways are important signal pathways involved in various cellular processes, including inflammatory reactions and immunity, etc. [32]. Therefore, levels of MAPKs, including c-Jun N-terminal protein kinases 1/2 (JNK1/2), p38, and extracellular-signal-regulated kinases 1/2 (ERK1/2), in cells infected with PCV2 and PRV alone or in combination were examined. The results showed that expression levels of the *p38α* gene and *ERK2* gene significantly decreased in all the virus-infected groups, including single- and co-infected groups, compared to that of the control group (Figure 8A,B). The expression levels of the *JNK1* gene were decreased in single- and co-infected groups compared with that of the control group (Figure 8C). On the contrary, both phosphorylated p38 (p-p38) and JNK1/2 (p-JNK1/2) were enhanced in all the infected groups (Figure 8D–F), whereas phosphorylated ERK1/2 (p-ERK1/2) were inhibited in all the infected groups, except the PCV2 alone group (Figure 8D,G). 

The above results indicate that single infection and coinfection of PCV2 and PRV can induce inflammation and immune responses by activating p38 and JNK1/2.

## 3. Materials and Methods

### 3.1. Cells and Virus

The porcine kidney cell line (PK-15, ATCC CCL-33) was previously purchased from American Tissue Culture Collection (ATCC, Manassas, VA, USA) and used in our lab [33,34]. PCV2 CC1 (GenBank accession: JQ955679) and PRV (Submission ID: 2573364) were isolated previously and stored in our lab [33,34]. 

PK-15 cells were cultured in Dulbecco’s modified Eagle’s medium (DMEM, Gibco, Therm Fisher Scientific, Shanghai, China) containing 5% fetal bovine serum (FBS, Clark Bioscience, Shanghai, China) at 37 °C, 5% CO_2_.

### 3.2. Viral Infection

Cells were plated in a 6-well plate for 12 h to reach 50–60% confluency and divided into 6 groups for virus infection. Group 1 was used as a control group (PK-15 group). Group 2 was infected with PCV2 alone (PCV2 group), and group 3 was infected with PRV alone (PRV group). Cells were infected with PCV2 (5 × 10^4^ genomic copies/μL, 600 μL/well) or PRV (10^3^ genomic copies/μL, 600 μL/well) alone for 1 h. Then, cells were washed with PBS twice and cultured in 2 mL fresh DMEM (5% FBS). After that, 36 h later, samples were collected and evaluated via real-time PCR or Western blotting.

Group 4 to group 6 were co-infected groups. In group 4 (PCV2-12h-PRV group), cells were infected with PCV2 (5 × 10^4^ genomic copies/μL, 600 μL/well) for 1 h, washed with PBS twice and cultured in 2 mL fresh DMEM (5% FBS). Then, 12 h later, cells were incubated with PRV (10^3^ genomic copies/μL, 600 μL/well) for 1 h and washed with PBS twice and cultured in 2 mL fresh DMEM (5% FBS) for 36 h. In group 5 (PRV-12h-PCV2 group), cells were infected with PRV (10^3^ genomic copies/μL, 600 μL/well) for 1 h, washed with PBS twice and cultured in 2 mL fresh DMEM (5% FBS). Once 12 h had elapsed, cells were incubated with PCV2 (5 × 10^4^ genomic copies/μL, 600 μL/well) for 1 h and washed with PBS twice and cultured in 2 mL fresh DMEM (5% FBS) for 36 h. Then, samples were collected and evaluated via real-time PCR or Western blotting. In group 6 (PCV2+PRV group), cells were co-infected with PCV2 (10^5^ genomic copies/μL, 300 μL/well) and PRV (2 × 10^3^ genomic copies/μL, 300 μL/well) for 1 h. Then, cells were washed with PBS twice and cultured in 2 mL fresh DMEM (5% FBS). After that, 36 h later, samples were collected and evaluated via real-time PCR or Western blotting.

### 3.3. Real-Time PCR

Virus genomic DNA was extracted using TIANamp Virus DNA/RNA Kit (Tiangen, Beijing, China) according to the manufacturer’s instructions. Then, real-time PCR was performed using primers PCV2-Q-F/R or PRV-Q-F/R (Table S1) to evaluate levels of virus genomic DNA [35,36]. 

Total RNA was extracted from mock and the virus-infected cells at 36 hpi using TRNzol Universal Reagent (Tiangen, Beijing, China), and reverse-transcribed in cDNA using FastKing gDNA Dispelling RT SuperMix (Tiangen, Beijing, China), according to the manufacturer’s instructions. Thereafter, expression levels of host genes were evaluated via real-time PCR with corresponding primer pairs (Table S1). Quantitative real-time PCR (qRT-PCR) and relative mRNA levels were performed using the 2^−ΔΔCt^ method, and mRNA levels were calibrated and compared with the cells that had undergone 36 h in PK-15.

### 3.4. Western Blotting

Total protein was extracted from mock and the virus-infected cells at 36 hpi. Briefly, cells were washed with PBS twice, digested with trypsin, and centrifuged at 1000 rpm for 5 min. The collected cells were lysed with 200 μL cell lysis buffer for Western and IP (Beyotime, Shanghai, China) on ice for 30 min, followed by centrifugation at 12,000 rpm, 4 °C for 20 min. The supernatant was transferred into a clean tube and quantified using an Enhanced BCA Protein Assay Kit (Beyotime, Shanghai, China), according to the manufacturer’s instructions. 

Western blotting was performed according to the protocol described by Ouyang previously [37]. Briefly, protein samples were separated via 10% SDS-PAGE and electro-transformed onto the PVDF membrane. Then, the membrane was blocked with 5% skim milk for 2 h at room temperature and incubated with primary antibody at 4 °C overnight. Thereafter, the membrane was incubated with HRP-labeled Goat Anti-mouse IgG (H+L), HRP-labeled Donkey Anti-Goat IgG(H+L), or HRP-labeled Goat Anti-rabbit IgG (H+L) (1/2000, Beyotime, China) for 90 min at room temperature. Subsequently, the protein band was developed using an ECL kit (WLA006a, WanleiBio, Shenyang, China) and examined via Bioanalytical Imaging System c600 (Azure Biosystems, Dublin, CA, USA). 

Interferon beta/IFNB, IFN-γ, P-IΚBα (Ser32 Ser36), IΚB-α, IL1α, IL6, TNFα, NLRP3, Cox-2, P38, and p-p38 (Thr180/Tyr182) were purchased from Wanleibio (rabbit, Shenyang, China). Anti-STAT1 Antibody (rabbit) and Anti-β-Actin Antibody (mouse) were purchased from Boster Biological Technology (Wuhan, China). NFκB p65 Polyclonal Antibody and Phospho-NFκB p65 (Thr276) Polyclonal Antibody were from Therm Fisher Scientific (rabbit, Shanghai, China). IL28/29 (H-1) (mouse), JNK (FL) (rabbit), and p-JNK (Thr 183/Tyr 185, goat) were purchased from Santa Cruz Biotechnology (Dallas, TX, USA). Interferon-alpha 1 Ab, p-STAT1, and IRF9 Antibody were purchased from Affinity Biosciences (rabbit, Changzhou, China). ERK p44/42 MAPK (Erk1/2) (137F5) Rabbit mAb and Phospho-p44/42 MAPK (Erk1/2) (Thr202/Tyr204) (D13.14.4E) XP^®^ Rabbit mAb were from Cell Signaling Technology (Boston, MA, USA). IL1B Antibody was from Cusabio Biotech (rabbit, Wuhan, China). Rabbit Anti-IRF7 antibody was from Bioss (rabbit, Beijing, China).

Grayscale values were calculated and analyzed with Image-Pro Plus 6.0.0.260 (Media Cybernetics, Rockville, MD, USA). Grayscale values were recorded three times. The relative expression of each protein was expressed as mean grayscale values of target protein/mean grayscale values of β-actin. The average value of the relative expression is shown below each lane.

### 3.5. Statistical Analysis

All data are shown as representative results or means from at least three independent experiments ± SD. The statistical analysis was conducted using GraphPad Prism 8.0.2 (San Diego, CA, USA) with a One-way or Two-way analysis of variance (ANOVA). The resulting *p* < 0.05 was a statistically significant difference. 

## 4. Discussion

Coinfection of viruses and/or bacteria has been frequently reported in the field [1,2,4,6,10,38,39,40]. Recent reports showed that coinfection of PCV2 and PRV is relatively high in the field [1,6,10], which may aggravate the diseases and reduce the vaccine protection rate, thus causing the virus to become widespread. Previous studies have shown that PCV2 can inhibit the production of IFN-α and IFN-β [15,41], and herpesvirus can escape the cellular immune responses induced by IFNs and the JAK/STAT pathway [42,43,44]. In this study, the changes in immune-related signal pathways and inflammatory factors caused by PCV2 and PRV infection alone or in combination were evaluated. The results indicate that the expressions of IFN-γ, IFN-λ1, IRF7, and ISG15 were enhanced during the coinfection of PCV2 and PRV. However, coinfection of PCV2 and PRV can prevent the cascade of IFN signals by inhibiting the JAK/STAT pathway (Figure 4), suggesting coinfection of PCV2 and PRV has stronger immunosuppression on the IFN-JAK/STAT pathway than that of the PCV2 and PRV infection alone (Figure 2, Figure 3 and Figure 4). Based on this, the suggestion is that viral infection enhances the initiation of the immune state of the host cell, but evades the immune response by inhibiting the IFN-JAK/STAT pathway. Notably, coinfection of PCV2 and PRV also promotes the expression of IFN-λ1, which is consistent with previous reports that type III IFN can be used to inhibit influenza and coronavirus infections [45,46], suggesting that type III IFN is a promising antiviral agent against PCV2 and/or PRV infections. 

Furthermore, the results in this study also demonstrated that infection of PCV2 and PRV alone or in combination can stimulate the NF-κB signaling pathway by enhancing the levels of phosphorylated p65 and iκB (Figure 5). Both phosphorylated p38 and JNK1/2 were enhanced in all the infected groups (Figure 8). These results indicate that NF-κB and MAPK pathways mediate antiviral immune responses and the activation of inflammatory factors in PCV2 and PRV coinfection, which is consistent with previous results of PCV2 or PRV infections alone [15,18,47,48,49]. These results also confirm our above results, that is, the up-regulation of IFN-λ1, ISG15, IRF9, IL6, and IL1β may be related to NF-κB and MAPK pathways.

TNFα is an important pleiotropic cytokine, which is involved in host immunity, inflammation, and apoptosis [19,50,51]. TNFα can be used as a pro-inflammatory factor to stimulate strong inflammatory reactions and immunosuppression and maintain immune homeostasis by limiting the degree and duration of the inflammatory process [50,51]. We found in this study that PCV2 infection alone enhanced the expression levels of the *TNFα* gene more than that of the control group (Figure 6). Compared with PCV2 infection alone, coinfection of PCV2 and PRV decreased the expression levels of the *TNFα* gene. However, the protein levels of TNFα in all infected groups were inhibited, and the inhibitions of TNFα in the coinfection groups were slightly stronger compared with single infections. These results indicate that the virus may inhibit the aggravation of cellular inflammation by inhibiting the expression of TNFα. However, compared with PCV2 infection alone, coinfection of PCV2 and PRV decreased the levels of the *IL1α* gene but increased the level of the *IL6* gene and *IL1β* gene. Compared with PRV infection alone, coinfection of PCV2 and PRV increased the levels of the *IL1α* gene and *IL1β* gene but decreased the level of the *IL6* gene. Furthermore, infection of PCV2 and PRV caused inflammasome proteins NLRP3 and COX2-mediated cellular inflammatory reaction, and the coinfection of PCV2 and PRV aggravated the inflammation (Figure 7). These results demonstrate that coinfection of PCV2 and PRV may cause more severe inflammatory reactions and immune disorders. 

**Figure 9:**
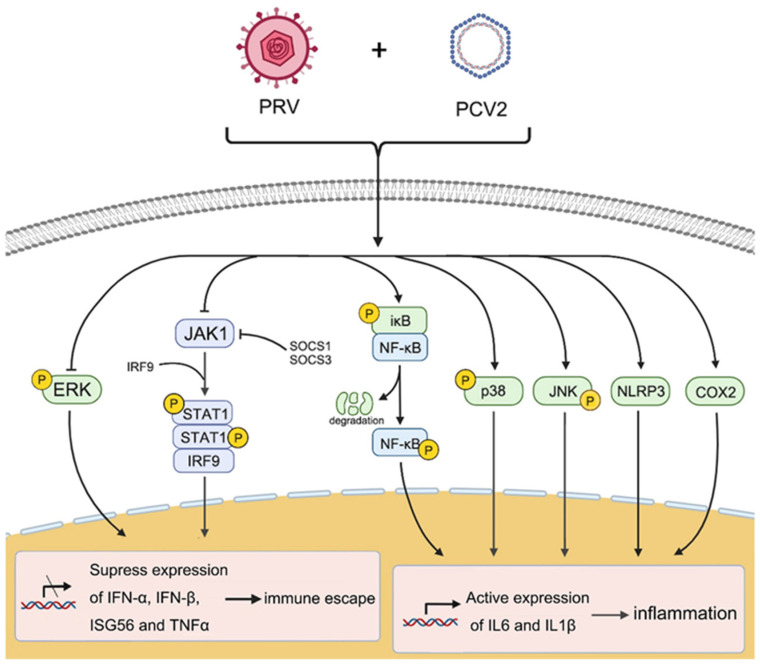
Proposed immune and inflammatory reactions caused by coinfection of PCV2 and PRV. Arrow (→), enhance; T line (⊥) means inhibition.

Therefore, the coinfection of PCV2 and PRV shows a stronger ability to induce inflammation and immunosuppression through several cellular pathways, which may be one of the reasons for the aggravation of infected animals. The coinfection of PCV2 and PRV can promote the activation of NF-κB, JNK, p38, and NLRP3 pathways, thus enhancing the expression of IL6 and IL1β, and finally potentiating cellular inflammation. Meanwhile, PCV2 and PRV also inhibit the expression and signal transduction of the IFN-β, TNFα, and JAK/STAT pathways, thus inhibiting inflammation and immune responses (Figure 9). It is worth mentioning that the changes in the expression levels of the inflammatory factors and cytokines also imply that there is a regulatory mechanism to maintain dynamic balance in the interaction between host cells and viruses, which not only has antiviral inflammation and immune responses but also has a protective mechanism to inhibit excessive inflammatory damage. Further research on the exact mechanism of PCV2 and PRV coinfection on host cell inflammation and the immune response is in progress in our lab. 

## 5. Conclusions

In conclusion, the results of this study showed that the coinfection of PCV2 and PRV can promote the activation of NF-κB, JNK, p38, and NLRP3 pathways, thus enhancing the expression of IL6 and IL1β, and finally potentiating cellular inflammation. Meanwhile, PCV2 and PRV also inhibit the expression and signal transduction of the IFN-β, TNFα, and JAK/STAT pathways, thus inhibiting immune responses. These results indicate that the regulations of cellular antiviral immune responses and inflammatory responses mediated by NF-κB, JAK/STAT, MAPK, and NLRP3 pathways, contribute to immune escape of PCV2 and PRV and host antiviral responses, which may be of great value for the research and control of infection and related diseases of PCV2 and PRV. 

## Data Availability

All data generated or analyzed during this study are included in this published article.

## Supplementary Materials

The following supporting information can be downloaded at: https://www.mdpi.com/article/10.3390/ijms23084469/s1. References [35,36] are cited in the supplementary materials.
