# Supplementary material for: Coinfection of Porcine Circovirus 2 and Pseudorabies Virus Enhances Immunosuppression and Inflammation through NF-κB, JAK/STAT, MAPK, and NLRP3 Pathways
Source: Int J Mol Sci. 2022 Apr 18;23(8):4469. doi: 10.3390/ijms23084469 (PMC9029761; doi:10.3390/ijms23084469)
Supplement: Supplementary file 1 [file ijms-23-04469-s001.zip › ijms-1679993-supplementary.pdf]

Supplementary Information for

Coinfection of porcine circovirus 2 and pseudorabies virus enhances immunosuppression and inflammation through NF-κB, JAK/STAT, MAPK, and NLRP3 pathways

Xue Li#, Si Chen#, Liying Zhang#, Guyu Niu, Xinwei Zhang, Lin Yang, Weilong Ji, Linzhu Ren*

*** Correspondence:** Linzhu Ren
Email: renlz@jlu.edu.cn

This PDF file includes:

Figures S1 to S7
Tables S1
SI References

Supplemental figures

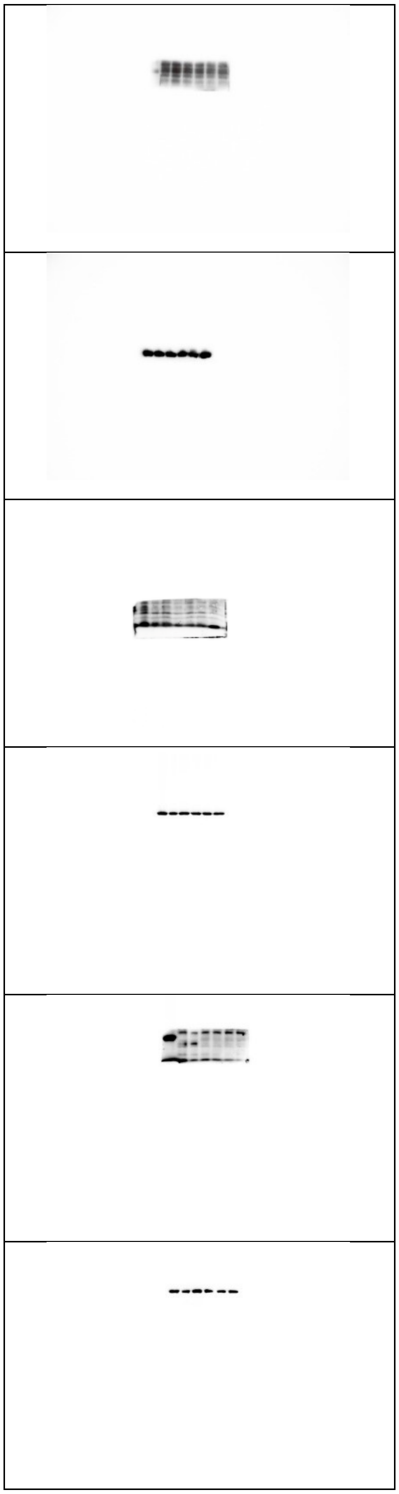

IFN-α

IFN-α β-actin

IFN-β

IFN-β β-actin

IFN-γ

IFN-γ β-actin

IFN-λ1

IFN-λ1 β-actin

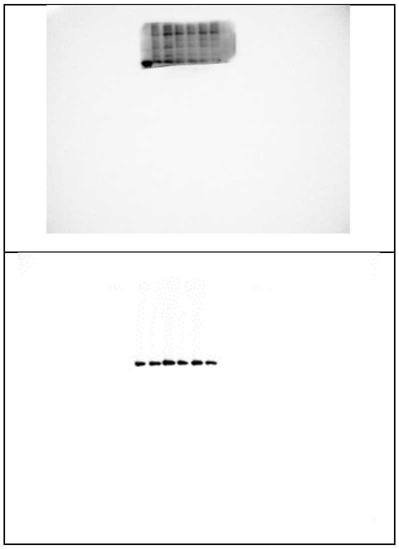

Figure S1: Original Western images used for preparing Figure 2.

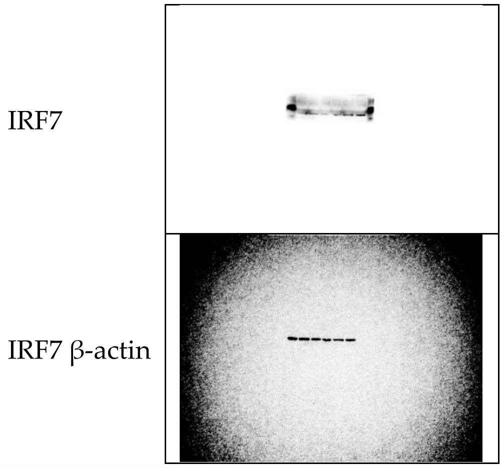

Figure S2: Original Western images used for preparing Figure 3C.

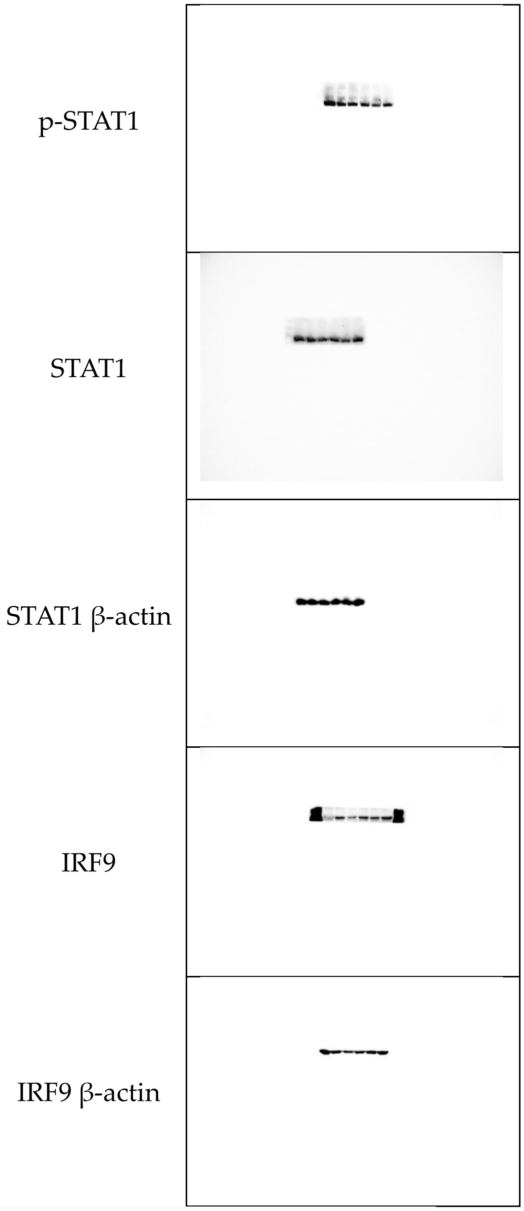

Figure S3 Original Western images used for preparing Figure 4E.

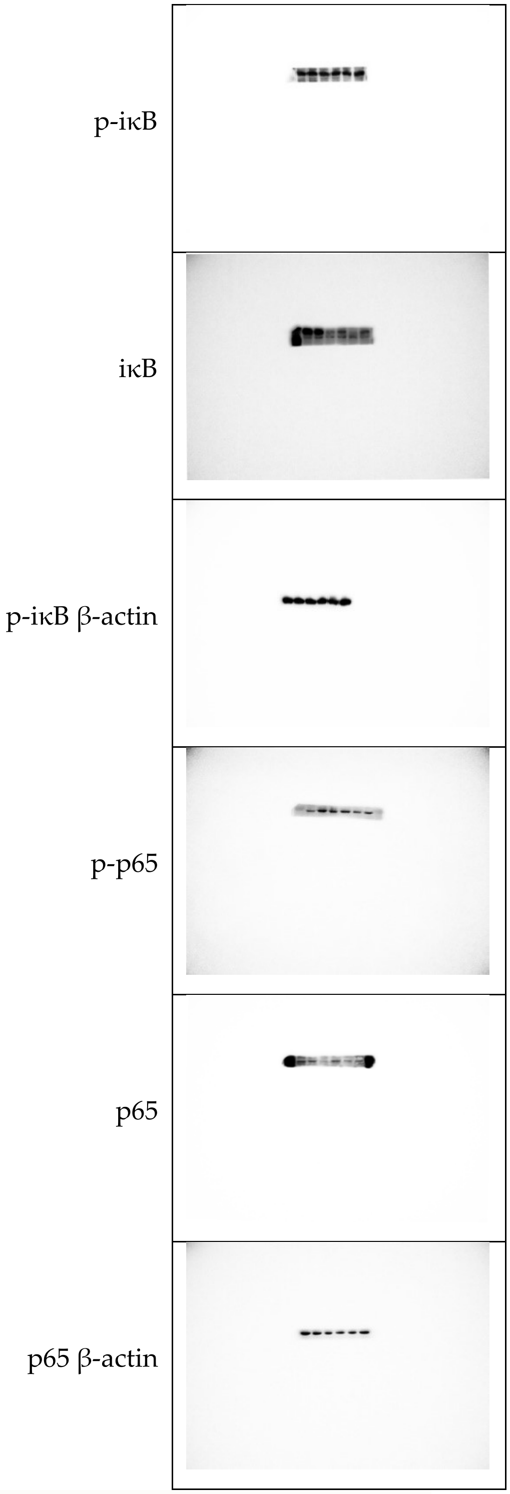

Figure S4 Original Western images used for preparing Figure 5C.

IL1α

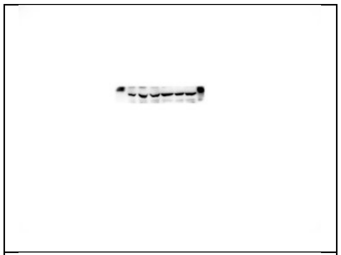

IL1α β-actin

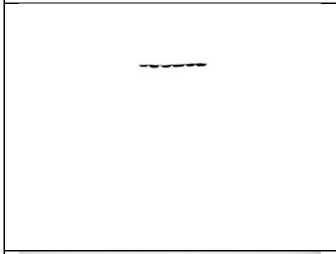

TNFα

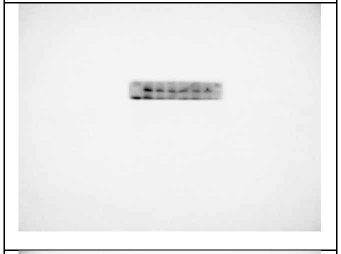

TNFα β-actin

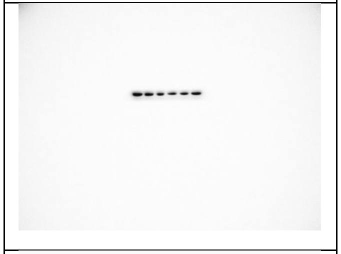

IL6

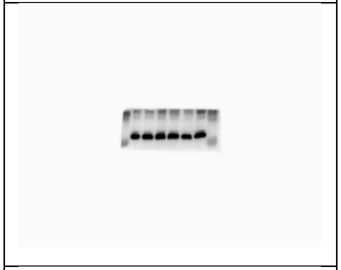

IL6 β-actin

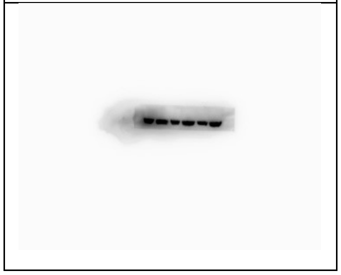

pro-IL1β

and IL1β-cleaved

IL1β actin

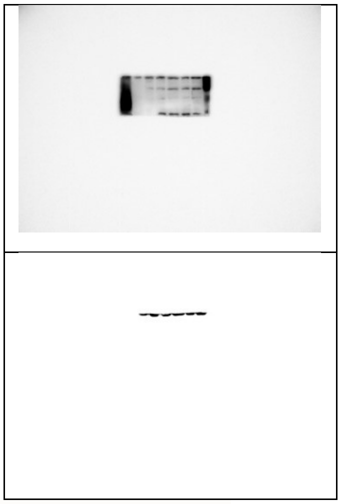

Figure S5 Original Western images used for preparing Figure 6E.

NLRP3

COX2

NLRP3, COX2 β-actin

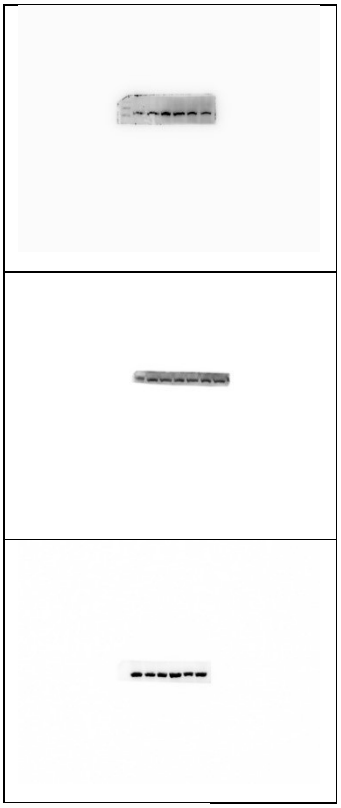

Figure S6 Original Western images used for preparing Figure 7C.

p-p38

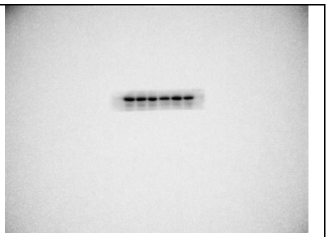

p38

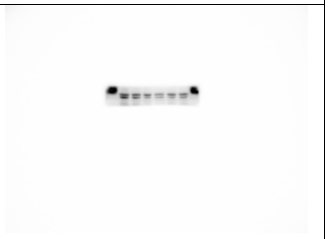

p-JNK1/2

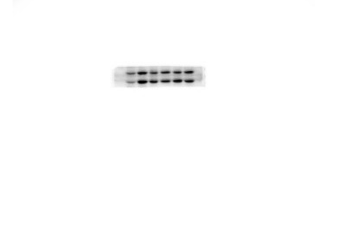

JNK1/2

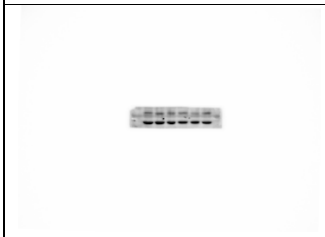

JNK1/2, p38 β-actin

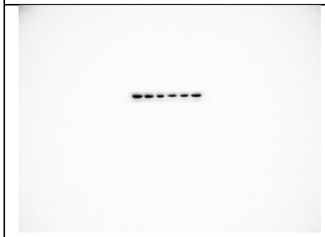

p-ERK1/2

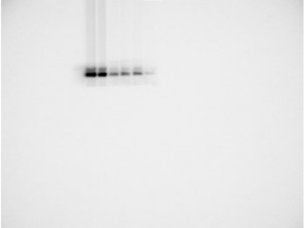

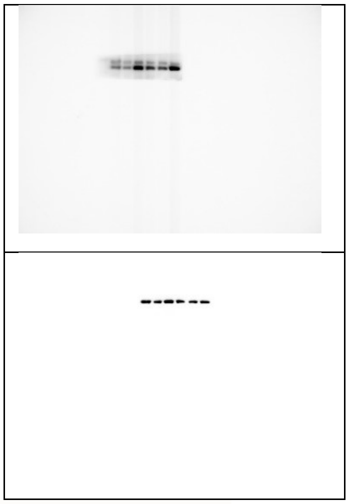

ERK1/2

ERK1/2 β-actin

Figure S7 Original Western images used for preparing Figure 8D.

Supplemental Tables

Table S1 Sequence of primers

Primer	Sequence (5′-3′)	GenBank No.	Reference
PCV2 Cap-Q-F	TGTAGTATTCAAAGGGCACAGAGC	NC_005148 (1,034..1,735)	(1)
PCV2 Cap-Q-R	CGGATATACTATCAAGAAAACCAC		
PRV gD-Q-F	GGTTCAACGAGGGCCAGTACCG	NC_006151 (121,075..122,277)	(2)
PRV gD-Q-R	GCGTCAGGAATCGCATCACGT		
GAPDH-F	GCCATCACCATCTTCCAGG	NM_001206359	(1)
GAPDH-R	TCACGCCCATCACAAACAT		
Pig ISG15-F	GGCAATGTGCTTCAGGATGG	NM_001128469	This study
Pig ISG15-R	CAGACCTCATAGGCGTTGCT		
Pig ISG56-F	GCCTCTCCTTTTAGCCACAGA	NM_001244363	This study
Pig ISG56-R	GCCCATGTTGCTGTTATGTCC		
Pig IL-1A-F	TGGGAGGACCAGGGTTACTG	NM_214029	This study
Pig IL-1A-R	GGTCCATCAGCAACTTAAAGGCA		
Pig TNFα-F	AACCTCAGATAAGCCCGTCG	NM_214022	This study
Pig TNFα-R	GGCATACCCACTCTGCCATT		
Pig IL-6-F	ATCCAGACCCTGAGGCAAAA	NM_214399	This study
Pig IL-6-R	AGGTGCCCCAGCTACATTAT		
Pig IL1β-F	ACCCTCTCCAGCCAGTCTTCATT	NM_214055	This study
Pig IL1β-R	TTGCAGCTGGATGCTCCCATT		
Pig IRF3-F	CCCACCTGGAAGAGGAATTT	NM_213770	This study
Pig IRF3-R	CTCCTGAGGTCACAAACTCATAG		
Pig IRF7-F	AGCCCTCTACCCCCATCTGT	NM_001097428	This study
Pig IRF7-R	AGCCAGTCTCCGAAGAGCAC		
Pig IRF9-F	GGAACTGGGTGGTGGAGCAA	NM_001078670	This study
Pig IRF9-R	CTCTTGTTGAGGGCACAGCG		
Pig RELA/p65-F	TGGTGGGAAAACACGGAGAG	NM_001114281	This study
Pig RELA/p65-R	ATGCCTCTGTCATTCGTGCT		
Pig IκBα -F	CCTGGTGTCGCTCTTGTTGA	NM_001005150	This study
Pig IκBα -R	GCTGCTGTATCCGAGTGCTT		
Pig Jak1-F	CCAGGCAAGAGTGCATAGAA	NM_214114	This study
Pig Jak1-R	GTGGTTCCAAAGCTCCATTTG		
Pig SOCS1-F	TCAGTGTGAAGATGGCTTCGG	NM_001204768	This study
Pig SOCS1-R	AGCTCGAAGAGGCAGTCGAA		
Pig SOCS3-F	ATCCCTCTGGTGTTGAGCCG	NM_001123196	This study
Pig SOCS3-R	AGTGGGGCATCGTACTGGTC		
Pig STAT1-F	CCCTGACGGTCTTATTCC	NM_213769	This study
Pig STAT1-R	ATAGTCGTTCCTCGCTCT		
Pig STAT3-F	TTCAAACACCTGACCCTG	NM_001044580	This study
Pig STAT3-R	TCAGCACCAGTAGAGGTTG		
Pig p38/MAPK14-F	GAAAGCAGGGACCTCCTTATAG	XM_003356615	This study
Pig p38/MAPK14-R	CGGATTCTCAAGACTCCATCTC		
Pig ERK2-F	ACCTCAGCAACGACCATA	NM_001198922	This study
Pig ERK2-R	AGACACTGAAACCCAACC		
Pig JNK1-F	AGTTGGATGAAAGGGAACACACA	XM_021073087	This study
Pig JNK1-R	ACTGCTGCACCTGTGCTAAA		
Pig NLRP3-F	ACCTGCAAAAACTGGGGTTGG	NM_001256770	This study
Pig NLRP3-R	TCACCCAGGTCATTGTTGCCT		
Pig COX2/PTGS2-F	GAGGTCTTTGGTCTGGTGCCT	NM_214321	This study
Pig COX2/PTGS2-R	CAGCCGTTCATCGTCCCATTC		

Reference

1. H. G. Shen *et al.*, Protective immunity against porcine circovirus 2 by vaccination with ORF2-based DNA and subunit vaccines in mice. *J Gen Virol* **89**, 1857-1865 (2008).
2. Z. Peng *et al.*, Pseudorabies virus can escape from CRISPR-Cas9-mediated inhibition. *Virus Res* **223**, 197-205 (2016).
